# Supplementary material for: Predictors of institutional delivery service utilization among women in Northern region of Ghana
Source: PLoS One. 2025 Jun 5;20(6):e0324328. doi: 10.1371/journal.pone.0324328 (PMC12140280; doi:10.1371/journal.pone.0324328)
Supplement: Attachment — Submitted filename: PONE-D-23-14896 Review comments.docx [file pone.0324328.s002.docx]

**PONE-D-23-14896 MANUSCRIPT REVIEWERS COMMENTS**

ABSTRACT

Stop first sentence at” morbidity and mortality”

There on free maternal health care but NHIS

Why did you not study the prevalence and predictors of home delivery? Some reasons why they delivered at home may still remind unknown.

Conclusion: What is “availability of skilled personnel”?

LINES 25-39: The comments above also apply to this section

LINES 47-48 When does a woman become A SINGLE MOTHER? Is it when she is pregnancy or after delivery.? Are they unmarried pregnant women? Teenagers? or their husbands travelled while they were pregnant?

LINES 54-73: These definitions are not necessary in the manuscript

LINE 76: ..in the “reproductive process”….this is not clear. You need to it up from the reference 1.

METHODS

LINES 122: Data collected from 10^th^ March- 2^nd^ April 2023 and compare to -lines 31: Jan 2022 to Jan 2023. Put the two sentences together so that it becomes clear what you want to do.

What is community chairman? Chairman of what?

LINE 163: Needs clarity. Respondents were recruited at the starting point and every fifth house was visited.

LINE 172: What about COVID 19? When you are collecting data in March- April 2023.

RESULTS IN THE TABLES

TABLES 1: Differences between divorced and single. Almost 38% (119) were single women in those rural communities yet 245 husbands had positive or negative attitudes as shown in table 4.

That makes total number of husbands 54 more than your study of 310 respondents. Are some of the women having more than a husband or what. In the same table 4, under husband’s occupation total is 320 also more than your study of 310 respondents.

Where from these errors? How do these errors affect your findings and conclusions?

DISCUSSION

LINES 317-344

How is the impact of pregnancy schools relevant in this study which was in a rural population?. Something you found in your study attracts them to health facilities so discuss that more.

CONCLUSION LINES 329-331: The major issue with the conclusion is that there is no clear understanding of who is a single mother.

The data from tables 1 & 4 are conflicting on marital status of the women, attitudes of the husbands and number of husbands in the various occupations listed.

REFEENCES

All references must conform to Journals referencing style/format

Some references are incomplete eg 22 and 23 are not complete
