# Supplementary material for: Predictors of institutional delivery service utilization among women in Northern region of Ghana
Source: PLoS One. 2025 Jun 5;20(6):e0324328. doi: 10.1371/journal.pone.0324328 (PMC12140280; doi:10.1371/journal.pone.0324328)
Supplement: Attachment — Submitted filename: Response to reviewers.docx [file pone.0324328.s004.docx]

Dear Editor and reviewers,

We appreciate all of the valuable comments from the reviewers of our work. We have revised our manuscript according to the reviewers’ comments, questions, and suggestions. We believe that the manuscript has been further improved.

Attached below are detailed responses to all the reviewer’s comments. The responses are shown in green and italicized. Please let us know if you still have any questions or concerns about the manuscript. We will be happy to address them, now promptly

**Reviewer 1 comments**:

Methods:
Page 7: Sample size determination and sampling process line 146- 150; details on how the 8 districts and two communities were randomly selected should be given. How was the sampling frame of 5 arrived at?

***Response***

*The details on how the sampling of the districts and regions has been included “In selecting the districts, the names of all 16 districts in the Northern region were written on pieces of paper and placed in a box. The box was shaken vigorously after which 8 pieces of paper were randomly picked from the box. The 8 names on the papers selected were the sampled districts used for the study. Communities in the selected districts ranged from 12 – 20. The communities were categorized under stratum A (list of urban communities in the district) and stratum B (list of rural communities in the district). In each of the districts, the names of communities in each stratum were written on pieces of paper and placed in a box, shaken and one piece of paper randomly selected from each stratum. This was repeated for each district until 16 communities were randomly sampled for the study” Line 187 - 195*

How was the southward direction decided to be followed first until there were no more houses before other directions from the start point?

***Response***

*Determining the direction to initiate sampling has been included “Considering the arrangement of building structures from the community leader’s or chief’s house, from the starting point, the next house was the K^th^ (house selection interval) house away in the southward or northward direction. For each community, the sampling interval (K) was determined as the total number of houses divided by the number of women to be sampled from the community. Houses were selected in this direction” Lines 215 – 220*

Discussion: Page 16 Line: 267-271; “ The disparity in the reported prevalence…………….” Explain why recruiting women who have given birth in the previous year would give such different prevalence from the women recruited from the reproductive age or expectant mothers?

***Response***

*The reason for the disparity in prevalence based on the participants recruited has been addressed “Whereas our study considered women who had given birth in the previous year the other studies recruited women within the reproductive age or expectant mothers as study participants. This implies that the reported prevalence in these studies was the preferred choice of place of delivery by the study participants whereas this study reported the actual prevalence of health facility delivery among the women after it had occurred” lines 573 – 575*

Any recent studies from the respective countries referenced in your study?

***Response***

*Yes, a finding from the 2017 – 2018 multiple indicator survey has been referenced in the study. line 563 - 565*

**Reviewer 2 comments**

ABSTRACT

Stop first sentence at” morbidity and mortality”

***Response***

*The sentence has been revised to effect the change see lines 29 – 30 “An increase in home delivery among expectant mothers may likely lead to high maternal and newborn morbidities and mortalities”*

There on free maternal health care but NHIS

***Response***

*The sentence has been addressed to include the NHIS, kindly see lines 30 - 31*

Why did you not study the prevalence and predictors of home delivery? Some reasons why they delivered at home may still remain unknown.

***Response***

*This has been stated as an area for further research to be conducted. kindly see lines 660 – 661 “Further research should be conducted to assess the factors influencing institutional delivery service or home delivery using qualitative approaches in the region”*

Conclusion: What is “availability of skilled personnel”?

***Response***

*The availability of skilled personnel has been revised to the presence of skilled health personnel at health facilities. Kindly see lines 49 - 50*

LINES 25-39: The comments above also apply to this section

LINES 47-48 When does a woman become A SINGLE MOTHER? Is it when she is pregnancy or after delivery.? Are they unmarried pregnant women? Teenagers? or their husbands travelled while they were pregnant?

***Response***

*The single mother as used in the study has been revised to unmarried pregnant women. kindly see line 64 – 66 “We recommend the Ministry of Health should develop well-defined care packages targeting unmarried pregnant women, negative health worker attitudes and negative community perceptions”*

LINES 54-73: These definitions are not necessary in the manuscript

***Response***

*The definitions of some indicators as used in this study has been deleted per the review comment*

LINE 76: ..in the “reproductive process”….this is not clear. You need to it up from the reference 1.

***Response***

*The phrase reproductive process as used in the background has been revised to “during child birth” kindly see line 71*

METHODS

LINES 122: Data collected from 10^th^ March- 2^nd^ April 2023 and compare to -lines 31: Jan 2022 to Jan 2023. Put the two sentences together so that it becomes clear what you want to do.

***Response***

*The two sentences have been kept together to provide more clarity as stated by the reviewer. Kindly see lines 153 – 154 “Data was collected from women who had a live birth between January 2022 and January 2023. The data collection period was March 10^th^ – April 2^nd^ 2023”*

What is community chairman? Chairman of what?

***Response***

*The word community chairman as used in the study has been revised to community leader. Kindly see line 214*

LINE 163: Needs clarity. Respondents were recruited at the starting point and every fifth house was visited.

***Response***

*How the participants were recruited has been revised to give more clarity to the work. kindly see line 215 – 222 “Considering the arrangement of building structures from the community leader’s or chief’s house, from the starting point, the next house was the K^th^ (house selection interval) house away in the southward or northward direction. For each community, the sampling interval (K) was determined as the total number of houses divided by the number of women to be sampled from the community. Houses were selected in this direction until there were no more houses in that direction, then houses were selected westward, then eastward in a zigzag fashion. Houses were visited in this sequence until the required respondents were obtained*”

LINE 172: What about COVID 19? When you are collecting data in March- April 2023.

***Response***

*The lack of COVID-19 data in the study has been explained as a limitation in the study. kindly see line 658 - 659*

RESULTS IN THE TABLES

TABLES 1: Differences between divorced and single. Almost 38% (119) were single women in those rural communities yet 245 husbands had positive or negative attitudes as shown in table 4.

That makes total number of husbands 54 more than your study of 310 respondents. Are some of the women having more than a husband or what. In the same table 4, under husband’s occupation total is 320 also more than your study of 310 respondents.

Where from these errors? How do these errors affect your findings and conclusions?

***Response***

*The differences in the single status and divorced as used in this study have been rectified. Kindly see the revision in line 303, Table 2. Also, the inconsistency in the reported numbers or frequencies has been revised. Table 2 and 4 has been revised to ensure consistency.*

DISCUSSION

LINES 317-344

How is the impact of pregnancy schools relevant in this study which was in a rural population?. Something you found in your study attracts them to health facilities so discuss that more.

***Response***

*As recommended, more details on factors such as community perception has been discussed and the statement on the impact of pregnancy schools in the study revised. Kindly see lines 612 - 656*

CONCLUSION LINES 329-331: The major issue with the conclusion is that there is no clear understanding of who is a single mother.

***Response***

*The single mother status as used in the conclusion has been revised to unmarried pregnant women. kindly see line 666*

REFEENCES

All references must conform to Journals referencing style/format

Some references are incomplete eg 22 and 23 are not complete

***Response***

*References have been revised to ensure consistency. Also, reference 22 and 23 has been completed. Kindly see lines 754 - 763*

**Reviewer 3 comments**

Introduction
Use of safe and effective delivery services including place of delivery is an important component of the Safe Motherhood concept. Hence, assessing predictors of institutional delivery could contribute to improving birth outcomes in the study setting.

***Response***

*The sentence provided by the reviewer has been incorporated into the introduction section of the manuscript abstract to provide more clarity. Kindly see lines 32 – 35*

**Minor revisions**

1. There are more recent estimates of global maternal mortality. The authors are advised to use these (eg Trends in maternal mortality 2000 to 2020: estimates by WHO, UNICEF, UNFPA, World Bank Group and UNDESA/Population Division. Geneva: WHO 2023).

***Response***

*The statement on maternal mortality related to pregnancy and childbirth has been revised to reflect the suggestions made by the reviewer. Kindly see 71*

2. Line 59: The definition given is that of maternal mortality ratio rather the rate.

***Response***

*The definitions of key terms used in the study have been deleted from the manuscript as suggested by other reviewers*

3. A few sentences need to be edited eg line 95 “…sustainable development goal…” should be written as “…Sustainable Development Goal...”. Lines 139 and 226 should be in the past tense; ie reside and perceive respectively should be in the past tense.

***Response***

*The revisions suggested have been affected. Kindly see line 124 (Sustainable Development Goal) and line 177 (resided) and line 373 (perceived)*

4. Some acronyms were used without defining them at the first instance eg CHPS, DHS, MIS, WHO etc. Acronyms/abbreviations used in Tables should all be defined below the table.

***Response***

*All acronyms used in the manuscript have been defined at its first usage. Kindly see the revision line 163 (Community-based Health Planning and Services - CHPS)*

5. Study setting: The authors should state the population of WRA in the region.

***Response***

*The population of women of reproductive age in the study area has been stated. Kindly see line 161*

6. Methods: The selected districts together with the number of women selected from each district should be stated.

***Response***

*The selected districts and the number of women selected from each district has been presented in Table 1, kindly see line 228*

7. Line 188: Although the authors stated that there were some exclusions due to missing data, there is no evidence to support this as the estimated sample size (310) is exactly the same as the number of women surveyed. It appears all women approached agreed and participated in the study. Otherwise, they should state the number of women who were excluded.

***Response***

*Information on the number of study participants who were approached and those who accepted to participate in the study has been stated by the authors. Kindly see lines 297 – 298 “Overall, 340 women were surveyed in the study and 91.2% (310/340) agreed to participate in the study”*

8. Ethical considerations: The authors should clearly describe how consent was obtained from minors (ie women <18 years of age)

***Response***

*How consent was obtained from minors in the study has been clearly stated in the ethical consideration section of the manuscript. Kindly see lines 279 – 280 “. Consent was obtained from the parents or husbands of women aged less than 18 years. Also, an assent form was obtained from the minors”*

9. Provide refs for the sampling procedure described on page 8 and pregnancy schools in Ghana (lines 317-324), which could be of interest to readers.

***Response***

*References have been provided for the sampling procedure, kindly see line 186. A reference has also been provided for the pregnancy schools stated in the manuscript. Kindly see 624.*

10. The authors should provide the mean age, standard deviation and the range.

***Response***

*The variable age was collected as a categorical variable, this makes it impossible to present the summary statistics as proposed by the reviewer*

11. In Table 4, there is no need to indicate ** against significant p-values as the authors have indicated in the methods section that p<0.05 will be considered statistically significant. I believe all stated p-values in the table will be interpreted in that context. The authors rounded off some ORs from Table 4 in the text (see lines 237-247). The authors should state ORs in the text as they are in the table for ease of reference. They also repeated some ORs with their 95% CIs within the same paragraph and in some instances the ORs were different (eg lines 239 and 244; and 237 and 246). As much as possible, the authors should avoid repeating results especially within the same paragraph. They can make their point without repeating the results.

***Response***

*Table 4 and its text interpretation has been revised to ensure consistency in the results reported. Always the repetition of ORs has been revised. The ** used in the table to indicate significant p values has been deleted. Kindly see lines 396 - 401*

12. The authors should provide areas for further research in the conclusion.

***Response***

*Areas of further research have been provided as suggested by the reviewers. Kindly see lines 660 – 661 “Further research should be conducted to assess the factors influencing institutional delivery service or home delivery using qualitative approaches in the region”*

**Major revisions**

1. My major concern with the study is the sample size and its estimation. I do not think a sample of 310 is representative enough of the entire population of women of reproductive age (WRA) in the Northern region of Ghana. What is the population of WRA in the region? The stated sample size only estimated a single proportion of home deliveries without incorporating the predictors of home deliveries (such as the proportions and measures of association eg ORs as used in this study). Besides, no adjustments were made for the community-based sampling technique eg could the design effect be modified?

***Response***

*The sample size was based on the number of deliveries recorded since our target population was women who had given birth a year prior to the study. So, although there is a large population of WRA in the region, the estimated number of deliveries in the region is much lower. Also, the design effect was adjusted for in the data analysis, where robust standard errors using the type of community as a clustering variable was conducted*.

2. In line 193 the authors stated the criteria for inclusion into the multivariable model as p<0.05 in the univariable analysis. Yet in Table 4, several variables with p>0.05 in the univariable analysis were included in the multivariable analysis (age group, religion, educational level, occupation, husband occupation). The multivariable analysis should be re-run without these covariates ie ensuring that only covariates which meet the inclusion criteria are included in the multivariable model.

***Response***

*The strategy used in selecting variables for the adjusted logistic regression analysis has been revised. Kindly see lines 270 – 271 “The adjusted logistic regression model's variables were selected using the stepwise regression approach”*

3. The authors should discuss the limitations of the study.

***Response***

*The limitations of the study have been discussed as suggested by the reviewer. Kindly see lines 657 – 661 “The study was not devoid of limitations; the quantitative nature of the study did not allow for the authors to explore the perception of women on the use of institutional delivery services. Also, data was not collected on COVID-19-related factors that could influence institutional delivery services. Further research should be conducted to assess the factors influencing institutional delivery service or home delivery using qualitative approaches in the region”*
