# Supplementary material for: Predictors of institutional delivery service utilization among women in Northern region of Ghana
Source: PLoS One. 2025 Jun 5;20(6):e0324328. doi: 10.1371/journal.pone.0324328 (PMC12140280; doi:10.1371/journal.pone.0324328)
Supplement: Attachment — Submitted filename: Response to Review Comments.docx [file pone.0324328.s005.docx]

Dear Editor,

We appreciate all the valuable comments on our work. We have revised our manuscript according to the editor’s comments. We believe that the manuscript has been further improved.

Attached below are detailed responses to all the comments. The responses are shown in yellow and italicized. Please let us know if you still have any questions or concerns about the manuscript. We will be happy to address them, now promptly.

**Editor**

We've checked your submission and before we can proceed, we need you to address the following issues:

**Comment 1**. Thank you for stating the following financial disclosure:

[The research was funded by the principal investigator].

Please state what role the funders took in the study. If the funders had no role, please state: "The funders had no role in study design, data collection and analysis, decision to publish, or preparation of the manuscript."

If this statement is not correct you must amend it as needed.

Please include this amended Role of Funder statement in your cover letter; we will change the online submission form on your behalf.

***Response****: The role of the funder has been revised and added to the cover letter per the recommendation of the editor. Kindly see the revised version of the cover letter with the statement “This research work was funded by the principal investigator who doubles as the corresponding author. The funder was involved in the design, data collection, data analysis, manuscript drafting, and the decision to publish the work”*

**Comment 2**. Please note that funding information should not appear in any section or other areas of your manuscript. We will only publish funding information present in the Funding Statement section of the online submission form. Please remove any funding-related text from the manuscript.

***Response****: The funding information as previously provided in the manuscript has been deleted from the document.*
