# Supplementary material for: Predictors of institutional delivery service utilization among women in Northern region of Ghana
Source: PLoS One. 2025 Jun 5;20(6):e0324328. doi: 10.1371/journal.pone.0324328 (PMC12140280; doi:10.1371/journal.pone.0324328)
Supplement: Attachment — Submitted filename: Response_to_reviewers_auresp_3.docx [file pone.0324328.s006.docx]

Dear Editor and reviewers,

We appreciate all of the valuable comments from the reviewers of our work. We have revised our manuscript according to the reviewers’ comments, questions, and suggestions. We believe that the manuscript has been further improved.

Attached below are detailed responses to all the reviewer’s comments. The responses are shown in green and italicized. Please let us know if you still have any questions or concerns about the manuscript. We will be happy to address them, now promptly

**Introduction**

**Comment**: The authors cited the 2019 facility delivery rate in Ghana. Can they look at the current rate from the Ghana Demographic Health Survey and argue from there very well?

**Response**: Data from the 2022 Demographic Health Survey has been used as recommended “

According to the 2022, demographic health survey in Ghana, 86% of live births in the 2 years preceding the survey delivered in a health facility. This implies more than 10% of children are still delivered outside the health facility setting” kindly see page 3, lines 61 - 66

**Comment**: Can they also state the regional institutional delivery rate in their argument, especially in the problem statement

**Response**: The regional institutional delivery rate has been added the problem statement as suggested “The Northern region of Ghana continues to experience one of the lowest institutional delivery rates in the country, with only 70.3% of births occurring in health facilities (DHS 2022). Surveys conducted in Brong Ahafo region and Chereponi district of northern Ghana, among 138 and 440 women respectively, revealed health facility deliveries ranged from 38 – 52% (4,5).” Kindly see page 3, lines 65 - 68

**Comment**: The study gap is not very clear. The authors should clearly state some of the articles in the area, especially in Ghana and the Northern part of the country, and their gap.

**Response**: The articles and their gaps have been included as suggested “Also, published studies from the region on institutional delivery service utilisation are mostly descriptive studies or focus on particular districts (12,13) and do not thoroughly investigate the correlates of institutional delivery service utilisation at the regional level” kindly see page 5, lines 93 - 96

**Comment**: The authors should indicate the rationale of the study. That piece is missing in the introduction.

**Response**: The rationale for the study has been included in the introduction “Despite the established consequences of deliveries outside a health facility among WRA, there is a dearth of knowledge on the prevalence and predictors of health facility delivery in the Northern region. Also, published studies from the region on institutional delivery service utilisation are mostly descriptive studies or focus on particular districts (12,13) and do not thoroughly investigate the correlates of institutional delivery service utilisation at the regional level. Understanding the factors influencing health facility deliveries in the Northern region is crucial for designing targeted interventions to improve maternal health outcomes. This study aims to identify the prevalence and predictors of institutional delivery service utilization at the regional level, providing evidence-based insights that can guide policymakers in formulating strategies to increase health facility deliveries and ultimately reduce maternal and neonatal mortality in the region.” Kindly see page 5, lines 90 - 100

**Comment**: There should be a theory for the study.

**Response**: The theoretical framework of the study has been included “The theoretical framework of the study will be guided by the Health Belief Model (HBM) and elements from Andersen's Behavioral Model of Health Services Use. These models offer a combined approach to understanding how individual beliefs, social context, and access to resources influence institutional delivery. This framework will guide the study in identifying key predictors of service utilization and provide a basis for designing targeted interventions that address specific barriers and motivators identified in the Northern region of Ghana.” Kindly see page 5, lines 103 - 106

**Methodology**

**Comment**: I believe some districts may have more communities than others, so selecting 16 communities from each district would bias the sampling; how did the authors deal with districts with more communities?

**Response**: A stratified sampling approach grouping the communities into urban and rural areas was used to enable us to understand the problem in both urban and rural areas “The communities were categorized under stratum A (list of urban communities in the district) and stratum B (list of rural communities in the district). In each of the districts, the names of communities in each stratum were written on pieces of paper and placed in a box, shaken and one piece of paper randomly selected from each stratum.” Kindly see page 7, lines 156 - 159

**Comment**: Can the authors state the lowest and highest number of communities for the districts selected?

**Response**: The number of communities in the districts selected has been stated as suggested by the reviewer “Communities in the selected districts ranged from 20 – 35. The communities were categorized under stratum A (list of urban communities in the district) and stratum B (list of rural communities in the district). In each of the districts, the names of communities in each stratum were written on pieces of paper and placed in a box, shaken and one piece of paper randomly selected from each stratum.” Kindly see page 7, lines 156 - 159

**Comment:** The authors indicated that the kth interval used in selecting households can be specific on the number.

**Response**: The kth interval varied based on the number of households in each of the communities. We calculated the kth interval for each community

**Comment**: This statement, “*For each community, the sampling interval (K) was determined as the total number of houses divided by the number of women to be sampled from the community,*” and another statement, “*A respondent was recruited at the starting point, and every fifth house starting from the southward direction was visited*” are conflicting statements. This statement needs to be well explained. Does this mean that the sampling interval was used differently in each community? The authors should add a supplementary sheet on how the sampling was done concerning the communities and the selection of houses.

**Response**: The kth interval varied based on the number of households in each of the communities. We calculated kth interval for each community. Also the statement on the kth household and the fifth household has been revised. Kindly see page 8, lines 170 - 180

**Commen**t: There should be a section on the measurement of variables. Clearly describe how the dependent variable and the independent variables were measured.

**Response:** “Dependent variable: The dependent variable in the study was the place of delivery in the most recent birth. The variable was binary (Health facility/Home).

Independent variables: The independent variables were divided into three groups, sociodemographic characteristics of the participants, health facility-level factors, and community-level factors. The sociodemographic characteristics included occupation, religion, marital status, educational level, income level, husband education, and husband occupation. Health facility-level factors include the availability of a health facility, the attitude of health workers, the presence of health workers at post, and the distance to the nearest health facility. The community-level factors included the availability of TBAs in the community, community perception, the attitude of partners towards health facility delivery, and availability of transportation to health facilities.” Kindly see page 7, lines 135 - 144

**Comment**: Authors should state when the data was collected (month and year).

**Response**: The data collection period, month and year have been added “The data collection period was March 10^th^ – April 2^nd^ 2023”. Kindly see page 6, line 115

**Results**

**Comment**: The authors should elaborate clearly on how the variables were selected for the logistic regression (Table 4). What were the criteria for reducing the variables to only 4?

**Response:** The adjusted logistic regression model's variables were selected using a forward stepwise variable selection approach. Only variables which were significant at the crude level were presented in the table

**Discussion**

**Comment**: Link the theory to the discussion.

**Response**: The discussions have been linked to the Health Belief Model (HBM) and elements from Andersen's Behavioral Model of Health Services Use. Kindly see page 16

**Recommendation**

**Comment**: “The recommendation of the study is not very strong. For instance, *“To increase health facility delivery, we recommend the Ministry of Health should institute a policy reform with a well-defined care package targeting unmarried pregnant women and health workers with a negative attitude and community perception.”* Can authors suggest a policy in that direction? They should add other recommendations, especially given the context that most of the communities are rural

**Response**: More recommendations towards improving facility delivery, particularly in the rural communities have been added “Also, the government should provide incentives for health workers working in rural communities.” Kindly see page 20, lines 353 - 354
